# ABCA1-mediated nascent HDL formation is precisely regulated by the plasma membrane cholesterol

**DOI:** 10.1016/j.jlr.2025.100762

**Published:** 2025-02-18

**Authors:** Fumihiko Ogasawara, Kazumitsu Ueda

**Affiliations:** Institute for Integrated Cell-Material Sciences (WPI-iCeMS), Kyoto University, Kyoto, Japan

**Keywords:** ABCA7, ABC protein, apolipoprotein A-I: Aster-A/GramD1a, ATP-binding cassette A1, high-density lipoprotein, cholesterol, phosphatidylcholine, SREBP cleavage–activating protein, sterol regulatory element–binding protein

## Abstract

Intracellular cholesterol transport is essential for maintaining cellular cholesterol homeostasis. ATP-binding cassette A1 (ABCA1) continuously moves cholesterol from the inner leaflet to the outer leaflet of the plasma membrane (PM) to maintain low inner leaflet cholesterol levels. When PM inner leaflet cholesterol levels exceed ER cholesterol levels, which are maintained at approximately 5 mol% by the complex of sterol regulatory element–binding protein (SREBP) and SREBP cleavage–activating protein (SCAP), Aster-A/GramD1a transports the excess cholesterol to the ER. Furthermore, ABCA1 removes excess PM cholesterol by promoting its efflux as nascent high-density lipoprotein (HDL) particles. Thus, cellular cholesterol homeostasis is maintained by the coordinated action of SCAP–SREBP, Aster-A/GramD1a, and ABCA1. While the regulation of SCAP–SREBP and Aster-A/GramD1a is well-understood, the mechanism governing ABCA1 activity remains less understood. In this study, we investigated the impact of PM cholesterol levels on ABCA1-mediated cholesterol and phosphatidylcholine (PC) efflux. Cells were treated with various concentrations of methyl-β-cyclodextrin (MβCD) or MβCD-cholesterol for 30 min to modulate PM cholesterol levels. We found that the initial velocities of both cholesterol and PC efflux were dependent solely on PM cholesterol levels, despite both being substrates for ABCA1. Intriguingly, when PM cholesterol levels dropped below 70% of the level observed in cells cultured in the presence of 10% FBS, both cholesterol and PC efflux ceased, even in the presence of abundant PC in the PM. Our findings suggest that ABCA1-mediated nascent HDL formation is precisely regulated to maintain optimal PM cholesterol levels.

Cellular cholesterol is highly regulated in mammalian cells. The central switch in controlling cellular cholesterol homeostasis is the complex of sterol regulatory element–binding protein (SREBP) and SREBP cleavage–activating protein (SCAP), which reside in the ER membrane (1, 2). The SCAP–SREBP system transcriptionally stimulates de novo cholesterol synthesis in the ER and cholesterol uptake from the blood as low-density lipoprotein (LDL) when needed and even suppresses cholesterol export by ATP-binding cassette A1 (ABCA1) through the action of microRNAs such as miRNA-33 and 148a (3, 4). The system is regulated by the ER cholesterol level, with approximately 5 mol% serving as the threshold, while 65%–90% of cell’s total cholesterol is held in the plasma membrane (PM) (5, 6). The mechanism by which the SCAP–SREBP complex in the ER senses changes in the PM cholesterol level has remained elusive.

Our previous work demonstrated that ABCA1 and Aster-A/GramD1a play key roles in this mechanism (7). First, ABCA1 continuously moves cholesterol from the inner leaflet to the outer leaflet of the PM to generate a cholesterol gradient between the two leaflets of the PM (8, 9). When the cholesterol concentration in the inner leaflet exceeds that in the ER, cholesterol is transported to the ER by Aster-A/GramD1a down the concentration gradient. Considering that ER cholesterol content is maintained at 5 mol% by the SCAP–SREBP system, the PM inner leaflet cholesterol, which is accessible to Aster-A/GramD1a, should be kept at a maximum of this concentration (5 mol%). Because ABCA1 and Aster-A/GramD1a are ubiquitously expressed in human body cells, they enable a response to local and temporal increase in PM cholesterol at the thousands of PM-ER contact sites for cholesterol internalization (7). This allows the SCAP–SREBP system to continuously sense the PM cholesterol level.

ABCA1 was originally identified as essential for high-density lipoprotein (HDL) formation through the genetic analysis of Tangier disease, characterized by plasma HDL deficiency (10, 11, 12). ABCA1 transfers cholesterol and phosphatidylcholine (PC) from the PM to apolipoprotein A-I (apoA-I), a lipid acceptor in the blood, to generate discoidal nascent HDL (13, 14). The two activities of ABCA1, nascent HDL generation and cholesterol transfer from the inner leaflet to the outer leaflet, are regulated separately (15). Therefore, ABCA1 not only moves cholesterol to the outer leaflet but also serves to eliminate excess PM cholesterol, maintaining an appropriate cholesterol concentration gradient across the PM bilayer. However, while the mechanism by which excess PM cholesterol is internalized by Aster-A/GramD1a is well-understood (7, 16), the regulation of excess cholesterol export by ABCA1 remains less understood. The ABCA1 expression level is transcriptionally regulated with cellular cholesterol levels (17, 18, 19), but this process takes several hours; thus, an alternative mechanism likely exists to finely control PM cholesterol levels. We investigated how nascent HDL generation by ABCA1 is regulated and found that it is precisely regulated by PM cholesterol levels. when PM cholesterol levels dropped below 70% of the level under normal culture conditions, both cholesterol and PC efflux ceased, even in the presence of abundant PC in the PM.

## Materials and methods

### Materials

Methyl-β-cyclodextrin (MβCD), cholesterol, and choline oxidase were purchased from Merck. Phospholipase D was obtained from Enzo Life Sciences. Cholesterol oxidase and horseradish peroxidase (HRP) were acquired from Oriental Yeast Co., Ltd. Amplex Red was purchased from Thermo Fisher Scientific. HRP-conjugated anti-mouse antibody was obtained from Santa Cruz Biotechnology. The monoclonal antibody against the extracellular domain of ABCA1 (MT-25) was generated as previously described (15).

### Cell culture

BHK/ABCA1 cells were kindly provided by the late Dr John Oram and Dr Chongren Tang of the University of Washington (20). In the cell line, protein expression can be strongly induced by adding the synthetic steroid mifepristone (GeneSwitch system, Thermo). HEK293/ABCA1 cells and HEK293/ABCA7 cells were established by introducing pIRES-puro3 vectors (TaKaRa) containing the cDNA of ABCA1 and ABCA7, respectively, into HEK293 cells, followed by selection with puromycin. The cell lines were grown in a humidified incubator (5% CO_2_) at 37°C in Dulbecco’s modified Eagle’s medium containing 10% heat-inactivated FBS.

### Lipid efflux

BHK/ABCA1 cells were seeded in 24-well plates at a concentration of 2 × 10^5^ cells/ml and incubated at 37°C for 24 h. The culture medium was then replaced with medium containing 0.02% BSA and 10 nM mifepristone, followed by further incubation for 24 h. For HEK293/ABCA1 and HEK293/ABCA7 cells, seeding was done at a concentration of 2.5 × 10^5^ cells/ml in 6-well plates, followed by incubation at 37°C for 24 h. The medium was then exchanged with medium containing 0.02% BSA and various concentrations of MβCD or MβCD-cholesterol and incubated at 37°C for 30 min. Cells were washed three times with medium and then incubated at 37°C in medium containing 50 μg/ml recombinant apoA-I.

### Lipid extraction

The culture medium was transferred to glass tubes, mixed with four-times volume of chloroform:methanol (2:1), and allowed to stand overnight at 4°C. The aqueous and intermediate layers were removed, and an equal volume of Milli-Q water to the original culture medium was added. The mixture was centrifuged at 3000 rpm for 10 min at 4°C using a HITACHI T4SS31 rotor, and the aqueous and intermediate layers were removed again. The chloroform/methanol layer was air dried at 40°C, and the lipid was re-dissolved by adding Hank’s Balanced Salt Solution (HBSS) containing 0.01% Triton X-100 and 0.5 mM cholic acid.

### Cholesterol and PC quantification

For cholesterol quantification, 50 μl of lipid extraction solution was transferred to a 96-well black plate and incubated at 37°C for 30 min with HBSS containing 4 U/ml cholesterol oxidase, 4 U/ml HRP, and 200 μM AmplexRed. For PC quantification, 40 μl of lipid extraction solution was transferred to a 96-well black plate and incubated at 37°C for 30 min with HBSS containing 1 mM CaCl_2_, 10 U/ml phospholipase D, 0.01% Triton X-100, and 0.5 mM cholic acid. Subsequently, 50 μl of HBSS containing 2 U/ml choline oxidase, 4 U/ml HRP, and 200 μM AmplexRed was added and incubated at 37°C for 30 min. The fluorescence intensity (excitation wavelength 535 nm, emission wavelength 590 nm) was measured using a microplate reader (Cytation 5; BioTek). Since the same number of cells was inoculated into each well and the 30-min treatment with MβCD or MβCD-cholesterol did not impact cell growth, PC and cholesterol values (ng/well) were not normalized. In fact, values exhibited minimal variation between wells.

### Measurement of cellular cholesterol content

BHK/ABCA1 cells were seeded in 48-well plates at a concentration of 2 × 10^5^ cells/ml and incubated at 37°C for 24 h. The culture medium was replaced with medium containing 0.02% BSA and 10 nM mifepristone, followed by further incubation for 24 h. Medium containing 0.02% BSA and various concentrations of MβCD or MβCD-cholesterol was then added, and the mixture was incubated at 37°C for 30 min. Cells were washed three times with phosphate buffered saline without CaCl_2_ and MgCl_2_ (PBS), harvested with Cell Dissociation Buffer (Thermo Fisher Scientific), and resuspended in PBS. Lipids were extracted from the cell suspension following the same procedure as described above and dissolved in 2-propanol. Cholesterol and PC were quantified using the Cholesterol E-Test Wako (Fujifilm) and Phospholipid C-Test Wako (Fujifilm), respectively. Cell counts were corrected by calculating the ratio of cholesterol to PC.

### ELISA

BHK/ABCA1 cells were seeded in 96-well plates at a concentration of 5 × 10^4^ cells/ml and incubated at 37°C for 24 h. The culture medium was replaced with medium containing 0.02% BSA and 10 nM mifepristone, followed by further incubation for 24 h. Medium containing 0.02% BSA and various concentrations of MβCD or MβCD-cholesterol was then added, and the mixture was incubated at 37°C for 30 min. Cells were washed three times with medium and incubated with anti-ABCA1 antibody (MT-25) and HRP-conjugated anti-mouse antibody-containing medium for 30 min at room temperature. After washing three times with PBS, cells were incubated with reaction solution [0.03 M citric acid, 0.04 M sodium hydrogen phosphate, 0.03% hydrogen peroxide, and 0.05% 2,2′-azino-bis(3-ethylbenzothiazoline-6-sulfonic acid ammonium salt)] for 10 min. Cells were lysed by adding 30 μl of 10% SDS, and absorbance at 420 nm was measured using a microplate reader (Cytation 5; BioTek).

### Statistical analysis

Statistical significance between two groups was evaluated using an unpaired *t* test. For three or more groups, evaluation was done using the Tukey test after one-way ANOVA.

## Results

### PM cholesterol levels affect both cholesterol and PC efflux by ABCA1

**Fig. 1:**
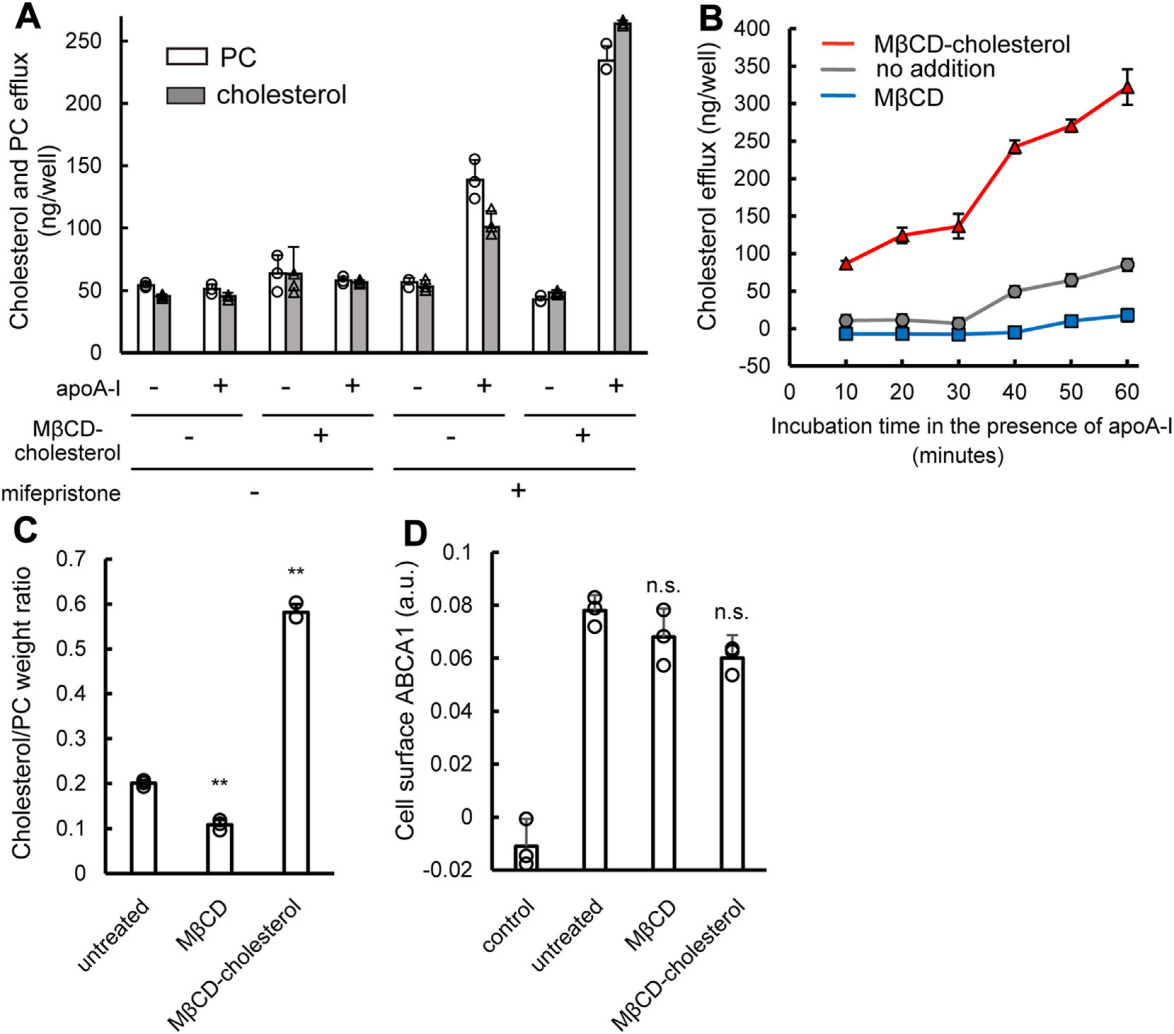
PM cholesterol levels affect both cholesterol and PC efflux by ABCA1. A: Cholesterol and PC efflux. BHK/ABCA1 cells were treated with or without 0.5 mM MβCD-cholesterol for 30 min at 37°C in the absence or presence of mifepristone. The cells were then incubated in the medium containing apoA-I for 1 h. The amounts of cholesterol (triangles, gray bars) and PC (circles, empty bars) in the medium were measured. Experiments were performed in triplicate. Values are means ± S.D. B: Time-dependence of cholesterol efflux. BHK/ABCA1 cells were treated with (gray line) or without 5 mM MβCD (blue line) or with 0.5 mM MβCD-cholesterol (red line) for 30 min at 37°C. The cells were then incubated in the medium containing apoA-I for the indicated time. The amount of cholesterol in the medium was measured. The graph shows the difference between samples incubated with apoA-I and a 10-min negative control (without apoA-I). Experiments were performed in triplicate. Values are means ± S.D. C: Cellular cholesterol/PC weight ratio. BHK/ABCA1 cells were treated with or without 5 mM MβCD or with 0.5 mM MβCD-cholesterol for 30 min at 37°C. To normalize the number of cells, the amount of cellular PC in the same sample was measured and expressed as the weight ratio of cholesterol to PC. Experiments were performed in triplicate. Values are means ± S.D. ∗∗*P* < 0.001 versus untreated. D: Cell surface ABCA1. BHK/ABCA1 cells were treated with or without 5 mM MβCD or with 0.5 mM MβCD-cholesterol at 37°C for 30 min, and then the cell surface ABCA1 content was measured by ELISA using the monoclonal antibody against the extracellular domain of human ABCA1 (15). Control is the value of the sample absent the primary antibody. Experiments were performed in triplicate. Values are means ± S.D. n.s. *P* > 0.05 versus untreated.

To investigate if PM cholesterol affects nascent HDL generation by ABCA1, we used BHK/ABCA1 cells in which the exogenous expression of human ABCA1 can be induced because stable and high expression of ABCA1 can alter cellular cholesterol metabolism. ABCA1 exports cholesterol and PC to generate nascent HDL in the presence of extracellular lipid acceptors, such as apoA-I, a major HDL component. To examine the immediate cellular response to an increased cholesterol level, the cells were treated with 0.5 mM MβCD-cholesterol for 30 min. They were then incubated with apoA-I for 1 h. Cholesterol (gray bars) and PC (empty bars) were exported into the medium in the presence of apoA-I from cells in which ABCA1 expression was induced with mifepristone (Fig. 1A). The MβCD-cholesterol treatment significantly increased cholesterol and PC efflux, namely nascent HDL generation, by ABCA1. ApoA-I–dependent lipid export was not observed even after MβCD-cholesterol treatment in cells in which ABCA1 expression was not induced. This observation suggests that ApoA-I–dependent lipid export is solely mediated by ABCA1 and that BHK cells have negligible endogenous ABCA1 expression. Additionally, we observed a linear increase in cholesterol efflux by ABCA1 for 1 h regardless of MβCD-cholesterol treatment, and MβCD-cholesterol treatment significantly amplified this increase (Fig. 1B, red and gray lines). Conversely, when the PM cholesterol level was lowered by MβCD-only treatment, cholesterol efflux by ABCA1 decreased (Fig. 1B, blue line). These results suggest that lipid export during the first hour in the presence of apoA-I can be considered as the initial velocity of ABCA1-mediated lipid efflux. This allows us to analyze the immediate impact of increasing or decreasing PM cholesterol on this process.

Figure 1C demonstrates a substantial alteration in the weight ratio of cellular cholesterol to PC following either treatment for 30 min. MβCD-cholesterol treatment elevated this ratio from 0.2 to 0.6, while MβCD-only treatment diminished it to 0.1. This shift in the ratio signifies a change in the cholesterol level within the PM, as neither treatment impacted the cellular PC content. Furthermore, a linear increase in cholesterol efflux was observed for 1 h subsequent to the MβCD-cholesterol treatment (Fig. 1B). ELISA with the monoclonal antibody against the extracellular domain of human ABCA1 (15) indicated that there was no significant change in the protein level of ABCA1 on the cell surface with either treatment (Fig. 1D). These results suggest that increasing the cholesterol level in the PM enhances both cholesterol and PC export by ABCA1, while a cholesterol deficiency suppresses them.

### ABCA1 generates nascent HDL to maintain proper PM cholesterol levels

**Fig. 2:**
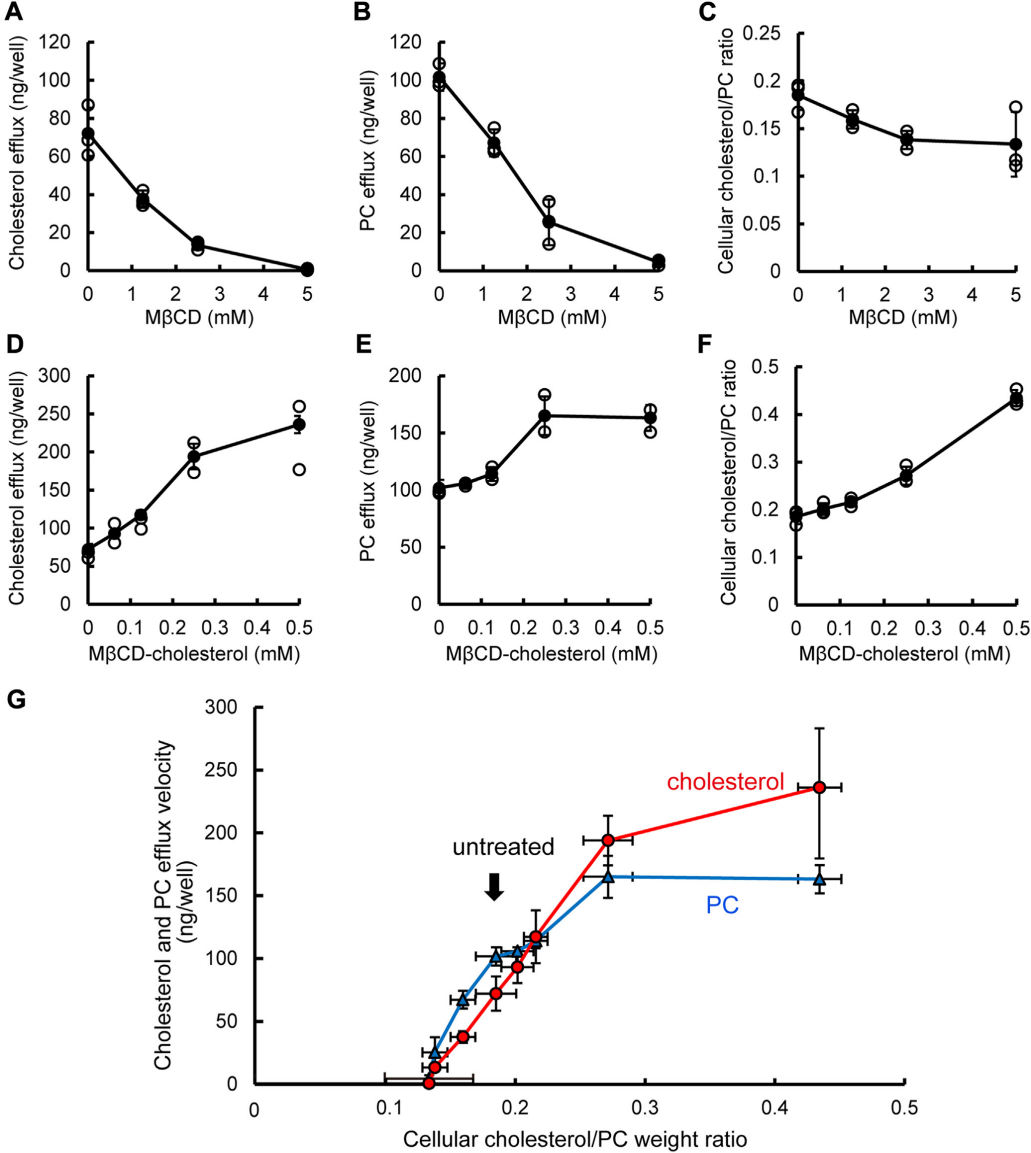
Both cholesterol and PC efflux velocities are precisely regulated by PM cholesterol. A, B, D and E: BHK/ABCA1 cells were treated with the indicated concentrations of MβCD or MβCD-cholesterol at 37°C for 30 min. The cells were then incubated in the medium containing apoA-I for 1 h. The amount of cholesterol and PC in the medium was measured. The graph shows the difference between samples incubated with apoA-I and a 1-h negative control (without apoA-I). Experiments were performed in triplicate. Values are means ± S.D. C, F: BHK/ABCA1 cells were treated with the indicated concentrations of MβCD or MβCD-cholesterol at 37°C for 30 min. Cells were washed three times with PBS and collected to measure the amount of cellular cholesterol. To normalize the number of cells, the amount of PC in the same sample was measured, and the cellular cholesterol is expressed as the ratio of cholesterol to PC. Experiments were performed in triplicate. Values are means ± S.D. G: The values of cellular cholesterol obtained in C and F were replotted on the horizontal axis, and the values of cholesterol and PC efflux obtained in A, B, D, and E are shown in the y-axis. The red circles and line represent cholesterol, and the blue triangles and line represent PC.

To further investigate the dependence of nascent HDL generation on PM cholesterol, BHK/ABCA1 cells were treated with various concentrations of MβCD or MβCD-cholesterol at 37°C for 30 min. ApoA-I–dependent cholesterol and PC efflux for 1 h were measured (Fig. 2A, B, D, E), and simultaneously, cellular cholesterol and PC contents were measured (Fig. 2C, F). Figure 2G illustrates the relationship between the cellular cholesterol/PC weight ratio and ABCA1-mediated cholesterol and PC efflux velocity.

The results show that the cholesterol efflux velocity (red line) increased linearly up to a cellular cholesterol/PC ratio of 0.27 following MβCD-cholesterol treatment. Similarly, PC efflux velocity (blue line) increased up to a cellular cholesterol/PC ratio of 0.27. Conversely, when the ratio was decreased by MβCD treatment, both cholesterol and PC efflux velocities decreased (Fig. 2G). And notably, when the ratio declined to a low of 0.13, both cholesterol and PC efflux ceased, despite the abundance of PC in the PM. Therefore, only cholesterol, and not PC, regulates ABCA1 activity, while both cholesterol and PC are substrates for ABCA1-mediated transport. These results indicate that ABCA1 generates nascent HDL to maintain proper cholesterol levels in the PM.

### PM cholesterol level recovers by the intracellular and extracellular cholesterol circulation

**Fig. 3:**
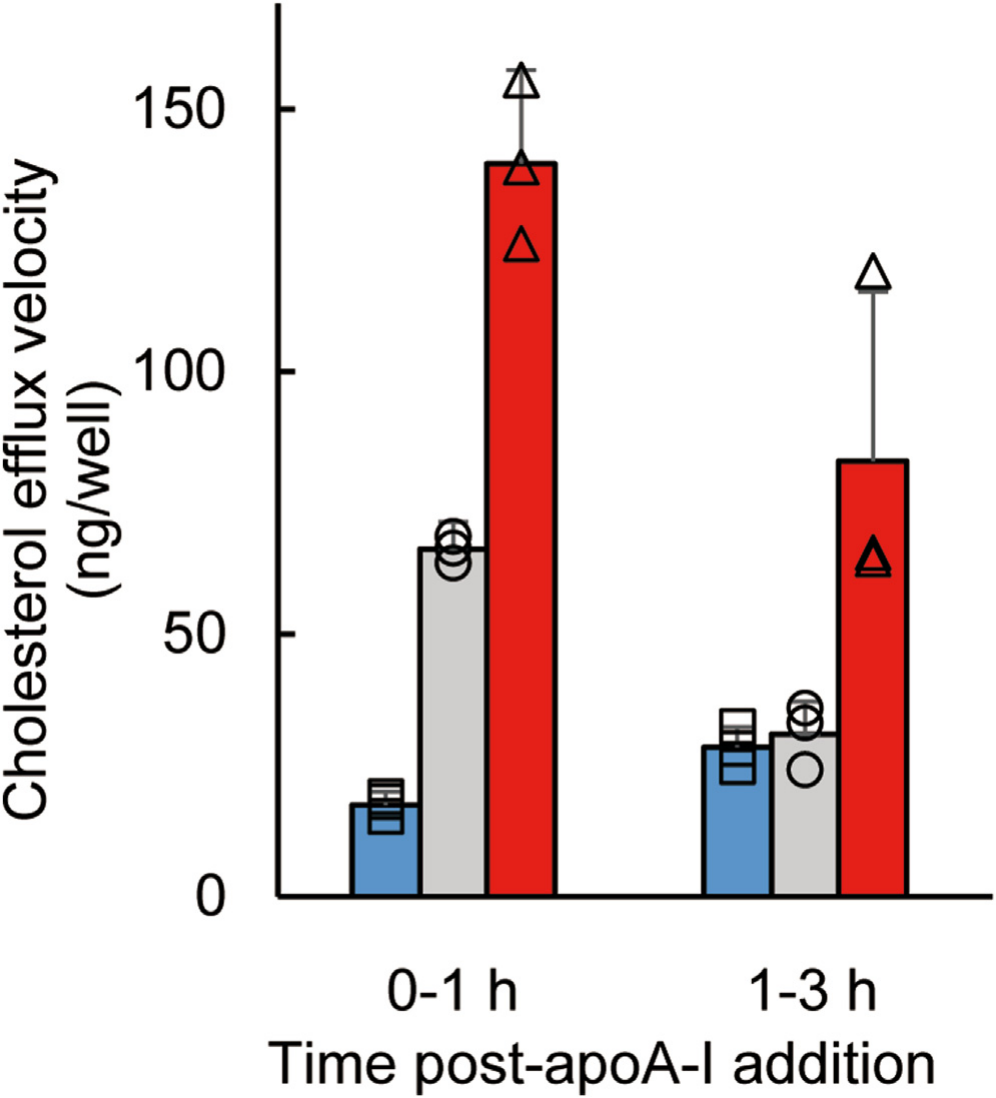
The velocity of cholesterol efflux by ABCA1 is quickly recovered overtime. BHK/ABCA1 cells untreated (circles, gray bars), treated with 5 mM MβCD (squares, blue bars), or treated with 0.5 mM MβCD-cholesterol (triangles, red bars) at 37°C for 30 min. Cells were washed three times with medium. The cells were then incubated in the medium containing apoA-I for 1 or 3 h. The amount of cholesterol released into the medium was measured. The cholesterol efflux velocity (1–3 h) represents the average rate of cholesterol efflux during the 2-h interval between 1 and 3 h. Experiments were performed in triplicate. Values are means ± S.D.

In this study, we measured cholesterol and PC efflux for 1 h. Since the efflux activity of ABCA1 depends on PM cholesterol levels, it was predicted that cholesterol efflux by ABCA1 would decrease as PM cholesterol decreased over time. Indeed, cholesterol efflux velocity between 1 and 3 h after adding apoA-I in untreated cells (gray bars) and cells treated with MβCD-cholesterol (red bars) decreased by 50% and 40%, respectively, compared to the first hour (Fig. 3). On the other hand, cholesterol efflux velocity between 1 and 3 h in cells treated with MβCD-only (blue bars) increased to the level of untreated cells (gray bars). These results suggest that the PM cholesterol level quickly recovers over time through intracellular and extracellular cholesterol circulation.

### PC efflux by ABCA7 is not affected by cholesterol

**Fig. 4:**
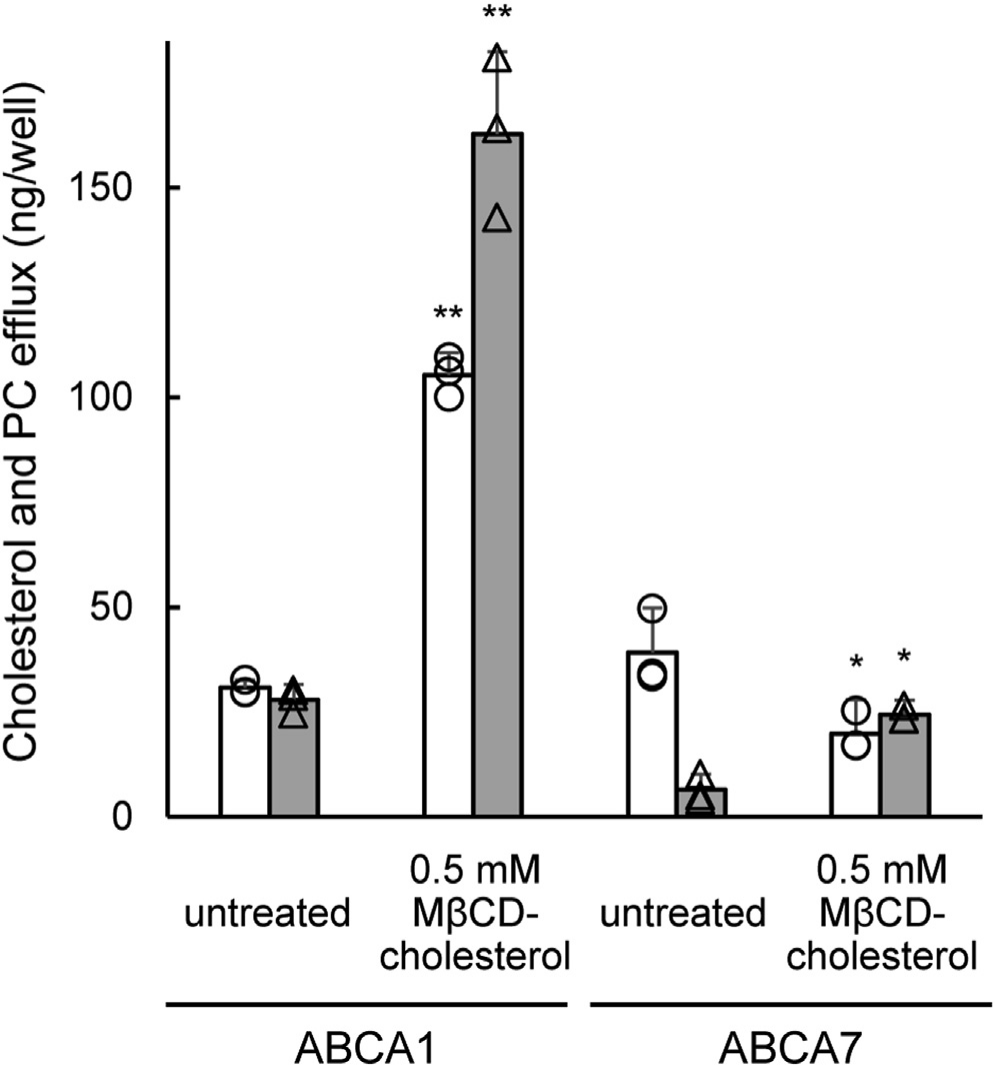
PC efflux by ABCA7 is not affected by cholesterol. HEK293/ABCA1 and HEK293/ABCA7 cells were treated with 0.5 mM MβCD-cholesterol at 37°C for 30 min. The cells were then incubated in the medium containing apoA-I for 1 h. The amount of cholesterol (triangles, gray bars) and PC (circles, empty bars) released into the medium was measured. The graph shows the difference between samples incubated with apoA-I and a 1-h negative control (without apoA-I). Experiments were performed in triplicate. Values are means ± S.D. ∗∗*P* < 0.001 versus untreated, ∗*P* < 0.05 versus untreated.

ABCA7 is an ABC protein with the highest amino acid sequence homology to ABCA1 (69% homologous, 54% identical) and exports PC onto apoA-I like ABCA1 (21, 22). ABCA7 is expressed predominantly in myelo-lymphatic tissues, but its physiological role is unclear. ABCA7 was also reported to export lysoPC (23), but its role in cholesterol transport is unclear. To examine whether the transport activity of ABCA7 is affected by the cholesterol level in the PM, HEK293/ABCA7 and HEK293/ABCA1 cells were treated with MβCD-cholesterol for 30 min and then incubated with apoA-I for 1 h. The amount of PC (circles, empty bars) exported by ABCA7 was slightly decreased by MβCD-cholesterol treatment, and the amount of cholesterol (triangles, gray bars) was slightly increased (Fig. 4). This observation is quite different from that of HEK293/ABCA1 cells, in which both PC and cholesterol efflux were greatly enhanced. These results indicate that the regulation of transport activity by cholesterol is not a general feature of lipid-transporting ABC proteins, but specific to ABCA1.

## Discussion

In this study, we investigated the effect of PM cholesterol levels on the velocity of cholesterol and PC efflux mediated by ABCA1. Cells were treated with various concentrations of MβCD or MβCD-cholesterol for 30 min to modulate PM cholesterol levels. Subsequently, the amounts of cholesterol and PC exported by ABCA1 over a 1-h period in the presence of apoA-I were measured, reflecting the initial velocity of cholesterol and PC efflux by ABCA1.

When the cellular cholesterol/PC ratio was increased by MβCD-cholesterol treatment, both cholesterol and PC efflux velocities increased in correlation with the ratio. Conversely, when the ratio was decreased by MβCD treatment, both cholesterol and PC efflux velocities decreased (Fig. 2G). These findings indicate that the rate of cholesterol and PC efflux mediated by ABCA1 is primarily determined by PM cholesterol levels, as PM PC level remained unchanged by these treatments, even though both cholesterol and PC are substrates for ABCA1-mediated transport.

ABCA1 loads PC and excess cholesterol in the PM onto apoA-I to generate discoidal bilayer nascent HDL. The cholesterol/PC weight ratio of nascent HDL produced from untreated cells was 0.71, and the molar ratio was 1.42 (Table 1). For nascent HDL produced from cells treated with 0.25 mM MβCD-cholesterol, the weight ratio was 1.18, and the molar ratio was 2.37. This indicates that nascent HDL particles produced from MβCD-cholesterol–treated cells contain 1.7-fold more cholesterol per particle compared to those produced from untreated cells. Duong *et al.* reported that ABCA1 generates two types of bilayer HDL particles (9 nm and 12 nm in diameter), and a large (12 nm) HDL particle can contain 1.6-fold more cholesterol compared to a small (9 nm) particle (14). It is possible that the size of nascent HDL particles produced by ABCA1 could be dependent on PM cholesterol levels.

**Table 1:** Relationship between cellular and nascent HDL cholesterol/PC ratios

MβCD-Treated Cell		Untreated Cell		MβCD-Cholesterol-Treated Cell	
Cellular	Nascent HDL	Cellular	Nascent HDL	Cellular	Nascent HDL
0.13 (0.26)		0.18 (0.36)	0.71 (1.42)	0.27 (0.54)	1.18 (2.37)

HDL, high-density lipoprotein; MβCD, methyl-β-cyclodextrin; PC, phosphatidylcholine. The cholesterol to PC weight ratios of MβCD (5 mM)-treated, untreated, and MβCD-cholesterol (0.25 mM)-treated cell and produced nascent HDL are presented. Their molar ratios (calculated using an average molecular weight of 776 Da for PC) are shown in parentheses.

When the cellular cholesterol/PC ratio was decreased by MβCD treatment, both cholesterol and PC efflux velocities not only decreased but also ceased when the cholesterol level fell below a certain threshold, despite the presence of abundant PC in the PM (Fig. 2G). This threshold was 0.13 in the cholesterol/PC weight ratio (0.26 in the molar ratio) (Table 1), which was only a 30% decrease from untreated cells. Collectively, these results indicate that ABCA1-mediated nascent HDL generation is precisely regulated by PM cholesterol levels to maintain appropriate cholesterol levels in the PM.

To maintain cholesterol level in the inner leaflet below that of the ER cholesterol (5 mol%), ABCA1 not only moves cholesterol to the outer leaflet but also eliminates excess cholesterol as nascent HDL, consuming a significant amount of energy. Is this effort solely for maintaining cellular cholesterol homeostasis? Liu *et al.* proposed (8) that the cholesterol concentration gradient between two leaflets of the PM allows cholesterol to function as an intramembrane signaling molecule. When cells are stimulated with the growth factor Wnt3, ABCA1 activity is temporarily suppressed, leading to a localized increase in cholesterol within the PM inner leaflet. As a result, a cytosolic signaling molecule, which has a cholesterol-binding domain, is recruited to the PM to stimulate the Wnt3 signaling. This signal transduction plays a crucial role in regulating cell division and differentiation. Given that the amino acid sequences of ABCA1, Aster-A/GramD1a, and SCAP are highly conserved among mammals, birds, and fish, the role of cholesterol as an intramembrane signaling molecule due to ABCA1-mediated asymmetric cholesterol distribution could be common among these animals. The evolution of the ABCA1–cholesterol signaling axis might have enabled vertebrates to develop complex developmental processes and sophisticated body plans.

Genetic variations in ABCA7 have been found to be strongly linked to Alzheimer's disease (24). ABCA7 shares a high degree of amino acid sequence to ABCA1, and, like ABCA1, it produces HDL-like particles in the presence of apoA-I (21, 22, 23). However, unlike ABCA1, ABCA7 exhibits minimal cholesterol export capacity. In fact, the regulation of ABCA7 is inversely related to that of ABCA1, as its expression is suppressed by high cholesterol levels and upregulated by low cholesterol levels (25, 26), suggesting that ABCA7 is not involved in maintaining cellular cholesterol homeostasis. Consistent with this, the present study revealed no significant increase in PC efflux when ABCA7-expressing cells were cholesterol loaded (Fig. 4). A slight increase in cholesterol efflux was observed, likely due to the nonspecific transfer of cholesterol to HDL-like particles produced by ABCA7. The absence of enhanced PC efflux in cholesterol-loaded ABCA7-expressing cells indicates that cholesterol-mediated regulation is not a universal property of lipid-transporting ABC proteins but is specific to ABCA1.

While cellular cholesterol homeostasis is highly regulated, as we demonstrated in this study, many mysteries remain (27). One such mystery is the regulation of ABCA1 in macrophages. At the early stages of foam cell formation, ABCA1 downregulation occurs through various mechanisms despite the increase in cellular cholesterol (28), leading to the subsequent accumulation of cholesteryl esters. Traditionally, foam cell formation has been primarily linked to cholesterol uptake. However, considering an alternative perspective, this process could be adapted to store fatty acids, an important energy source to support essential cellular functions. Understanding the pathological significance of various lipids, such as fatty acids and triacylglycerol, in addition to cholesterol, may be crucial for identifying novel therapeutic targets to limit foam cell formation (29).

In conclusion, our findings suggest that ABCA1-mediated nascent HDL generation is precisely regulated to maintain PM cholesterol levels appropriately. Further studies are necessary to fully understand the physiological importance of cholesterol and ABCA1-mediated HDL generation.

## Data availability

All data are contained within the article.

## Conflict of interest

The authors declare that they have no conflicts of interest with the contents of this article.
